# In Vitro Physicochemical and Pharmacokinetic Properties of Bevacizumab Dissolved in Silicone Oils Compared to Hydrogel-Substitutes and Porcine Vitreous Bodies

**DOI:** 10.3390/gels10080501

**Published:** 2024-07-28

**Authors:** Maximilian Hammer, Jonathan Herth, Lorenz Herbster, Manuel Ben Böhmann, Marcel Muuss, Ramin Khoramnia, Alexander Scheuerle, Walter Mier, Sabrina Wohlfart, Gerd Uwe Auffarth, Philipp Uhl

**Affiliations:** 1 University Eye Clinic Heidelberg, 69120 Heidelberg, Germany; mhammer@djapplelab.com (M.H.); ramin.khoramnia@med.uni-heidelberg.de (R.K.);; 2 Faculty of Biosciences, Heidelberg University, 69047 Heidelberg, Germany; 3 The David J Apple Laboratory for Vision Research, 69120 Heidelberg, Germany; 4 Institute for Pharmacy and Molecular Biotechnology, 69120 Heidelberg, Germany; 5 Department of Nuclear Medicine, Heidelberg University Hospital, 69120 Heidelberg, Germany

**Keywords:** silicone oil, bevacizumab, hydrogels, intravitreal injection, vitreoretinal surgery

## Abstract

Anti-VEGF agents, e.g., bevacizumab, are used in retinal surgery, while their interaction with silicone oils and novel hydrogels remains unclear. This study examines the in vitro pharmacokinetics of bevacizumab in silicone oil-filled eyes compared to various hydrogel replacements and the porcine vitreous body as well as its impact on the interface tension of silicone oils. An in vitro model filled with light or heavy silicone oil, porcine vitreous bodies, or hydrogels (alginate and polyethylene glycol (PEG)-based) was equilibrated with a balanced salt solution. Monitoring of bevacizumab in the aqueous phase was conducted for up to 24 h, and its effect on interfacial tension was studied. Significant differences in bevacizumab partitioning were observed across endotamponades after 24 h. In silicone oils, bevacizumab was found exclusively in the aqueous phase, while in the other endotamponades, it accumulated in the gel phase (96.1% in porcine vitreous body, 83.5% in alginate, and 27.6% in PEG-based hydrogel). Bevacizumab significantly reduced interfacial tension (40 to 8 mN/m), possibly enhancing silicone oil emulsification. The type of endotamponade heavily influenced the bevacizumab concentration in the aqueous. The vitreous body and replacement hydrogels likely serve as a drug reservoir, highlighting the need for in vivo studies to explore these interactions prior to clinical application.

## 1. Introduction

In cases of diseases requiring vitreoretinal surgery, the vitreous body must be removed from the vitreous cavity in order to safely access the retina. Replacing the vitreous body, e.g., in cases of rhegmatogenous retinal detachment or proliferative vitreoretinopathy, is challenging. Silicone oils have been used since their introduction in 1962 by Armaly and Cibis. Their surgical and rheological properties have been extensively studied and improved, leading to improved surgical instruments and heavier-than-water tamponades through the addition of semifluorinated alkanes [1,2,3,4]. However, their pharmacological properties in the context of additional drug administration (especially antibodies) have been poorly understood.

**Figure 1:**
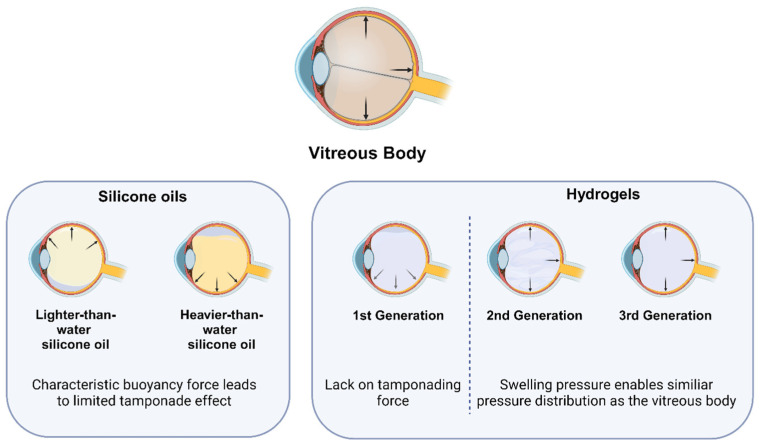
Characteristics of the tamponade effect of vitreous substitutes investigated in this study. Naturally, the vitreous body has an even pressure distribution within the posterior segment of the eye, which is why a complete tamponade effect occurs. The pressure distribution in silicone oil tamponades (**left**) depends on the applied density. Lighter-than-water silicone oils float on top of the water and cause a preferred pressure distribution on the upper segment in the vitreous cavity. On the other hand, heavier-than-water silicone oils have a higher density than water. The buoyancy force presses the silicone oil to bottom segment of the vitreous cavity. Those effects lead to an uneven pressure distribution when using silicone oils. Second- and third-generation hydrogels (**right**) however, show similar characteristics to vitreous body while the first-generation hydrogels lack tamponading force due to their low swelling pressure. They apply a uniform pressure across the total vitreous cavity.

The majority of the downsides of silicone oils are related to their tendency to emulsify. While certain risk factors, like intraoperative bleeding, have been established for emulsification, the influence of antibody solutions has never been explored. Additionally, hydrophilic vitreous body replacements like hydrogels have been preclinically developed over the past decades [5,6]. With the first pilot studies taking place in exceptional cases [7], information on the interaction of these hydrogels with simultaneous pharmacological therapies is crucial to allow the transition into clinical use (Figure 1).

Generally, recently developed hydrogels can be divided into four generations. Hydrogels of the first generation comprise viscous solutions of uncrosslinked polymers. Due to their short residence time, their application is limited to intraoperative use, where they are used as ophthalmological viscoelastic devices (OVDs) to stabilize the intraocular surgical field [8,9]. Further, they show an unsuitable swelling pressure and little tamponading force that is needed for replacements of the vitreous body. Hence, their tamponading effect is mainly based on their viscosity and specific gravity [9,10]. A wide variety of OVDs are currently in use, most of which are made of polymerized hyaluronic acid [11]. Further, multiple molecular weights of the polymers are used, differing also in their operative properties, dividing OVDs into cohesive and dispersive types [12,13].

To overcome the downsides of the first generation of hydrogels, second-generation hydrogels were developed. Longer residence duration as well as greater tamponading force could be achieved by using chemically crosslinked hydrogels [7,14,15,16]. These are formed in vitro by gelating the polymers with crosslinking solutions [15,17,18]. Second-generation hydrogels have been developed for different polymers, including hyaluronic acid [18], alginate [16], polyethylene glycol [19] and tetra-polyethylene glycol [17]. Surgical application of the gels through 23G trocar systems may cause fragmentation, which can alter the optical properties of the hydrogels [5]. Fragmentation may actually be aggravated by a trend towards even smaller-gauge vitrectomy surgery up to 27G [20,21]. Further challenges include confirmation of biocompatibility, which may be challenging [18,22,23,24].

Fragmentation of the hydrogels upon injection can be evaded by forming the hydrogel in situ. This is the concept of third-generation hydrogels. However, biocompatibility can become a major concern, as reactive molecules are injected. Thus, extensive testing of cytotoxicity is required. So far, many different gelation reactions have been developed, such as Schiff base reactions of polysaccharide-based polymers [25], aldehyde condensation of oxidated hyaluronic acid [26], and click chemistry with tetra-polyethylene glycol [17].

Another strategy of hydrogel formation is a physical crosslinking process. Therefore, the injected hydrogels are reversibly able to convert between a sol–gel state. This conversion can be triggered by temperature, pH change, or shear stress. The advantage of these fourth-generation hydrogels lies in their low toxicity and the independence of time reactions. By using polymer solutions instead of monomers, cytotoxicity by reactive substances or reactions is avoided. Furthermore, the surgeon or physician leading an operation is not restricted to time-dependent reactions, which carry the risk of early gelling reactions, in case the operation is unexpectedly delayed [10].

Many eyes requiring retinal surgery, especially in diabetic retinopathy, require the frequent administration of anti-VEGF therapeutics. Since the development of the anti-VEGF antibody bevacizumab and its introduction on the EU market in 2005, it revolutionized the treatment of neovascular retinal diseases [27,28].

Bevacizumab with its high polarity [29] and molecular weight (149 kDa), tends to dissolve in hydrophilic phases as it is also given under physiological conditions. To reduce stress and prevent antibodies and other biologicals from degradation, special pharmaceutical formulations are needed. A common strategy is to add surfactants, for instance, Tween 20, which is present in, e.g., Avastin. However, such additives lead to a different interaction between the biologicals and their surroundings [30].

Currently, there are little data available on the interaction of bevacizumab with lipophilic silicone oils and no data on the interaction of bevacizumab with vitreous body replacement hydrogel strategies [31]. The purpose of this study was to investigate the pharmacokinetics properties of bevacizumab in a previously established posterior segment model [4] filled with lighter-than-water and heavier-than-water silicone oils as well as hydrophilic vitreous body replacement strategies compared to the porcine vitreous bodies (PVB). Secondly, we assessed the impact of the bevacizumab solution as clinically applied on the interfacial tension between the aqueous phase and both types of silicone oil.

## 2. Results and Discussion

### 2.1. In Vitro Pharmacokinetics of Bevacizumab for All Tested Vitreous Body Substitutes

**Figure 2:**
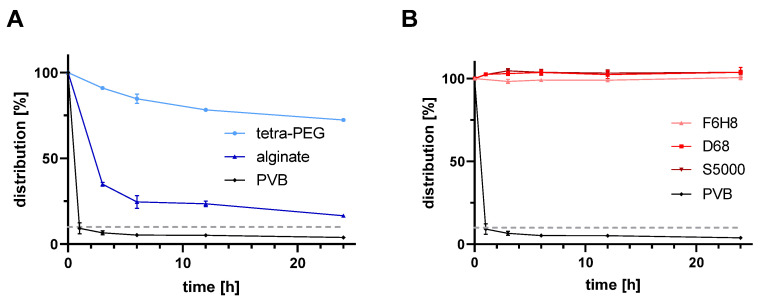
Concentration of bevacizumab in the aqueous phase within 24 h for (**A**) the tested hydrogels and (**B**) the silicone oils. The dotted line depicts a hypothetical concentration of bevacizumab for an equal distribution within the total volume in the vitreous cavity (2.3 mg/mL of 0.5 mL aqueous phase and 4.5 mL sample equivalent to 10% of the injected dose). The concentration of bevacizumab in the aqueous phase was measured via Nanodrop for the test samples except the porcine vitreous body. Measurement for the porcine vitreous body was conducted via ELISA. Abbreviations: tetra-PEG—tetra poly ethylene glycol hydrogel, alginate—alginate hydrogel, PVB—porcine vitreous body, D68—Densiron 68, S5000—Siluron5000; n = 6 (D68, S5000, PVB), n = 3 (F6H8, tetra-PEG, alginate).

The concentration of bevacizumab in the aqueous phase highly decreased for the hydrogel substitutes and the porcine vitreous body (*p* < 0.0001). Figure 2 displays the distribution of bevacizumab within the aqueous phase over 24 h. No distribution of bevacizumab into either light or heavy silicone oil was observed. F6H8, an additive in heavy silicone oil showed the same behavior as PDMS. Whereas the tPEG hydrogel showed a 20-fold increased bevacizumab concentration after 24 h (1.74 mg/mL), the alginate hydrogel only showed a 5-fold increased concentration (0.40 mg/mL) compared to the PVB (0.085 mg/mL). Within the test conditions, bevacizumab showed a preferred distribution into the PVB compared to an evenly distribution within the total volume (0.085 mg/mL PVB vs. 0.24 mg/mL for equal distribution in the total volume).

**Figure 3:**
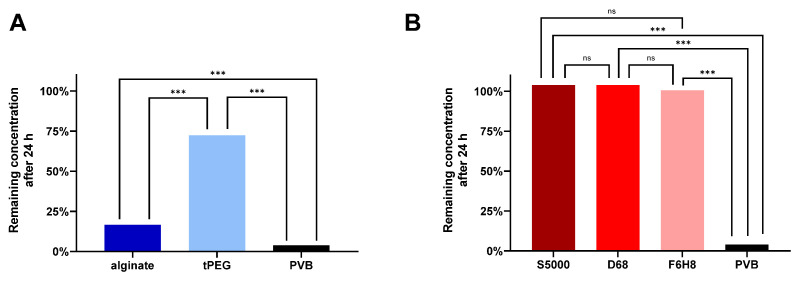
Remaining concentration of bevacizumab in the aqueous phase after 24 h of (**A**) the tested hydrogels and (**B**) the silicone oils in relation to the starting concentration. The concentrations of bevacizumab in the aqueous phases of the hydrogels and silicone oils were measured via Nanodrop, while the concentration in the PVB was measured via ELISA. Bevacizumab did not dissolve in silicone oil (S5000 = 103.74%, D68 = 103.75%, F6H8 = 100.58%, alginate = 16.53%, tPEG = 72.38% PVB = 3.86%). Abbreviations: tPEG—tetra polyethylene glycol hydrogel, alginate—alginate hydrogel, PVB—porcine vitreous body, D68—Densiron 68, S5000—Siluron5000; *** *p* < 0.0001, ns = not significant; n = 3 (alginate, tPEG, F6H8), n = 6 (S5000, D68, PVB).

Figure 3 compares the remaining concentration of bevacizumab after 24 h for all tested vitreous substitutes and the PVB. In comparison between the hydrogels and the PVB, both hydrogels incorporate significantly less bevacizumab than the PVB (*p* < 0.0001). The concentration of bevacizumab in the aqueous phase decreased to 16.5% for the alginate hydrogel (*p* < 0.0001), and to 72.4% (*p* < 0.0001) for the tPEG hydrogel. In contrast, the remaining concentration of bevacizumab within the aqueous phase of the PVB after 24 h was only 3.9% (10% would be expected at even distribution). All, the light and heavy silicone oils as well as 100% F6H8 (an additive in heavy silicone oil), showed no significant difference to baseline and compared to each other (F6H8 vs. D68 *p* = 0.1260; F6H8 vs. S5000 *p* = 0.1266; D68 vs. S5000 *p* = 0.9999; F6H8, D68 and S5000 vs. PVB *p* > 0.0001).

### 2.2. Influence of Bevacizumab on the Interfacial Tension between the Aqueous Phase and Light and Heavy Silicone Oils

**Figure 4:**
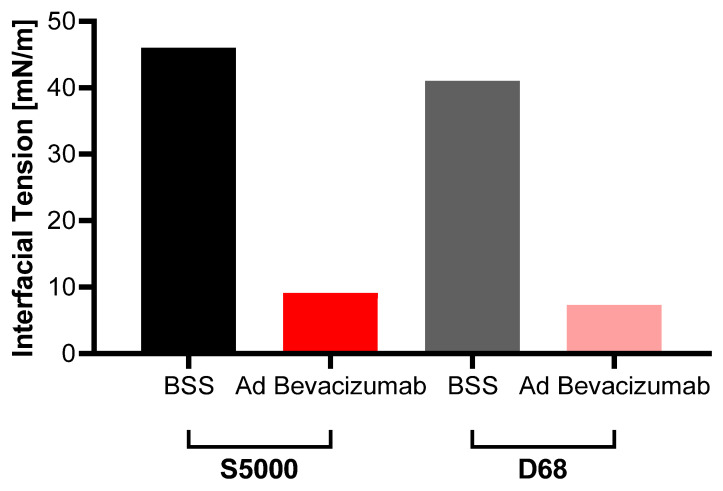
Interfacial tension of the silicone oils before and after injection of the bevacizumab solution. After adding bevacizumab to the aqueous phase, the interfacial tension of the silicone oils drops rapidly for S5000 (46 mN/m vs 9.1 mN/m) and D68 (41 mN/m vs. 7.3 mN/m). Abbreviations: D68—Densiron 68, S5000—Siluron5000; n = 1.

The interfacial tension between the aqueous phase and the light and heavy silicone oils decreased drastically after adding the bevacizumab solution to the aqueous phase (46 mN/m vs. 9.1 mN/m and 41 mN/m vs. 7.3 mN/m for Siluron 5000 and Densiron 68, respectively, Figure 4).

### 2.3. Discussion

#### 2.3.1. Summary of Results

To improve the understanding of the impact of modern intraocular endotamponades on intravitreal pharmacokinetics, we compared the distribution of bevacizumab in an in vitro model of various vitreous body substitutes in the eye. We observed a strong decrease in bevacizumab in the aqueous phase for all hydrogels and the PVB. In contrast, in the heavy and light silicone oils, no distribution of bevacizumab was observed. Furthermore, we investigated the effect of bevacizumab solutions in clinically relevant dosing on the interfacial tension of silicone oils. We found a drastic decrease in the interfacial tension regardless of the oil type.

#### 2.3.2. Silicone Oil and Intravitreal Injections

There are only a few studies available that address the interaction between intravitreal injections in silicone oil-filled eyes. Especially, regarding the physicochemical interactions between the drug and vitreous substitutes, a deeper understanding is inevitable. Xu et al. (2012) [32] investigated the pharmacokinetics of bevacizumab in the silicone oil-filled eye in pigmented rabbits and found an impact on the distribution of bevacizumab, which led to an altered profile of drug level in the ocular tissue [32]. However, in vivo studies observed many different influences on the pharmacokinetics simultaneously, such as clearance and diffusion mechanisms, without being able to differentiate.

The aim of this study was to examine the interaction of a clinically frequently used VEGF-agent solution with different vitreous substitutes in an established model. We observed that bevacizumab did not dissolve in heavy (Densiron 68) and light (Siluron 5000) silicone oils. Conclusively, there was no absorption of bevacizumab within the silicone oil. This is in line with in vivo studies exclusively examining light silicone oils: Xu et al. (2012) [32] observed that the concentration in the aqueous is increased compared to the vitreous body. They assume that this is caused due to a slower migration of bevacizumab from the silicone oil to the aqueous phase (24–72 h) compared to physiological conditions [32].

Recent studies investigated the distribution of other peptides, for instance, vancomycin in the established silicone-filled eye model [4]. It was shown that the concentration of vancomycin was also increased in the aqueous phase compared to the non-vitrectomized eye. This is probably caused by the decreased solubility of the peptide in the silicone oil compared to the physiological distribution into the vitreous body [33,34]. Applying our in vitro model, vancomycin was detected in concentrations of up to 53% in the aqueous phase of the tested light silicone oil. Meanwhile, bevacizumab was not soluble in silicone oil. The lipophilicity and therefore dissolution of peptides into the silicone oil is mainly driven by the moiety of polar groups: Vancomycin, a glycopeptide antibiotic, consists of a heptapeptide backbone with few hydrophilic groups, but also lipophilic aromatics that determine its characteristics. Bevacizumab, however, is a monoclonal antibody with a 100-fold higher molecular weight than vancomycin (149 kDa vs. 1.45 kDa). Thus, bevacizumab is more affine to the aqueous phase than vancomycin, as confirmed by our results. Nonetheless, both vancomycin and bevacizumab showed a higher drug concentration in the remaining aqueous phase in the silicone oil-filled eyes due to their hydrophilic affinity, regardless of whether their hydrophilic structures were small, moderately polar, or large and strongly hydrophilic.

#### 2.3.3. Interfacial Tension between Silicone Oil and Aqueous Phase

As biologicals and proteins are sensitive to hydrophobic surfaces, surfactants are added to their formulation to reduce physicochemical stress. We showed that the surface tension of the silicone oils rapidly decreased when adding the bevacizumab solution.

This is caused by the fact that the surfactants of the formulation accumulate at the interface between the hydrophilic and hydrophobic phases because of their amphiphilic character. By interacting with both interfaces, the intramolecular interactions are disrupted and interfacial tension is reduced. Conclusively, this leads to the formation of small emulsion droplets. Emulsification of silicone oils in vivo has been reported to occur over a wide range of time, which is why the exact process of emulsification is still not completely understood [35].

**Figure 5:**
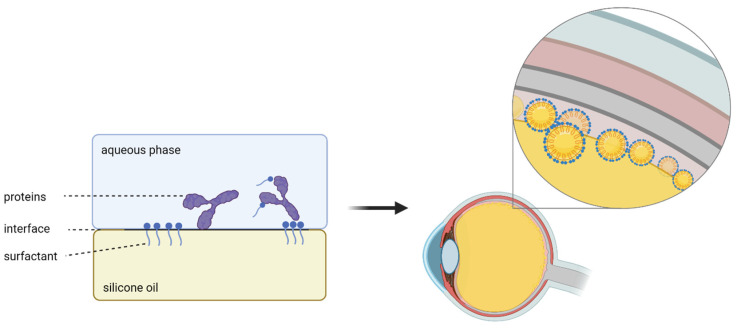
Schematic illustration of emulsification driven by surfactants. Surfactant additives, for instance, Tween 20, protect proteins from hydrophobic interfaces. They also reduce the interfacial tension and lead to enhanced emulsification which causes associated complications.

Several studies suggest that the emulsification of silicone oils is caused by eye movements [36,37]. This process is also facilitated and accelerated by biomolecules, for instance, proteins, as demonstrated by Nepita et al. (2020) [36]. They investigated the effects of γ-globulins and albumin on the interface tension in the absence of surfactants. The findings revealed a significant reduction in the interface tension of about 50% reaching 20 nM/m when the proteins were tested individually, and 17 nM/m when combined. It is noticeable that they used physiological concentrations of 25 g/L of γ-globuline and 50 g/L of albumin. In our experiment, we used a concentration of 2.3 g/L bevacizumab while the interface tension decreased even more drastically to 10 nM/m. This highlights the differences between the high effect of surfactants on the interface tension compared to those of proteins. In the silicone oil-filled eye the addition of surfactants is a major problem because it can lead to an aggravation (Figure 5). The added surfactants could increase emulsification rates, because of which postoperative problems like higher inflammation rates or higher intraocular pressure can occur. Of course, this is hard to prove in vivo as other factors, such as intraocular bleeding, are important additional causal factors.

#### 2.3.4. Bevacizumab and Hydrogels

Looking further, hydrogels from the second generation are incapable of forming droplets comparable to silicone oils, primarily due to their crosslinked structure. This study showed that bevacizumab preferentially distributes into the PVB. The concentration of recovered bevacizumab in the aqueous phase (3.9%) was lower than the theoretical concentration if distributed uniformly throughout the total volume (10% of the total amount used). From this point of view, PVB showed a potential storage capacity for proteins. In contrast to the silicone oils, the hydrogels, tested in this study, showed a decreased concentration of the antibody in the aqueous phase. Therefore, hydrogels could be potentially used as a depot form. For the tPEG hydrogel, 72.4% and for the alginate hydrogel only 16.5% remained in the aqueous phase.

**Figure 6:**
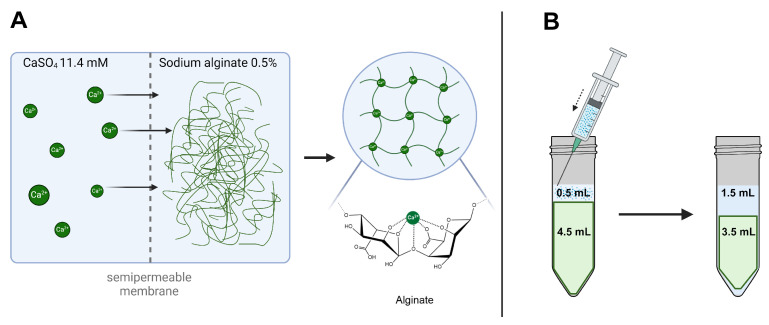
Synthesis and proposed syneresis of alginate hydrogel. (**A**) Alginate hydrogel is formed by dialyzing the sodium alginate in a CaSO_4_ 11.4 mM solution by complexation. (**B**) When adding the bevacizumab solution in BSS, the alginate hydrogel showed syneresis and diluted the concentration of bevacizumab.

The reduced concentration of bevacizumab in the aqueous phase of the alginate gel is caused by the syneresis of the hydrogel when adding the BSS solution. The alginate forms a hydrogel by complexation with calcium ions. This is achieved by dialyzing the alginate in a 11.4 CaSO_4_ solution, leading to an influx of calcium ions until the alginate is neutralized. When BSS is added to the alginate hydrogel, the ion concentration outside the alginate hydrogel is higher than inside the gel. Consequently, water molecules diffuse out of the gel due to an osmotic gradient resulting in a volume loss of the alginate hydrogel. On the other hand, the volume of the aqueous phase increases and leads to a diluted concentration of bevacizumab. These syneresis characteristics can potentially limit the application in a clinical setting. Apart from the light scattering effect after injecting the alginate gel through a 23G needle that we observed in another study [5], syneresis additionally causes a volume reduction of the hydrogel and leads to a lack of tamponading effect (Figure 6).

Hydrogels are crosslinked three-dimensional polymer networks. The tested anionic alginate hydrogel is naturally derived and formed of a linear polysaccharide polymer with alternating blocks of two monomers (β-mannuronic acid and α-guluronic acid). Due to the high hydrophilicity, the protein adsorption in alginate hydrogels is beneficial [38]. In studies of Xu et al. (2013) [39], the controlled release of bevacizumab of an alginate–chitosan copolymer hydrogel was already shown, but further improvements for an extended release are required [39]. This conducts the acceptable bevacizumab distribution in alginate hydrogels in this study. In contrast, the tested synthetic tPEG hydrogel incorporates less bevacizumab. An assumption for the weak incorporation in the tPEG hydrogel is, that the formed pores via a covalent thiol–maleimide-crosslinking are smaller than the hydrodynamic size of bevacizumab. A previous study showed that the pore size plays a crucial role in the hydrogel drug release of bevacizumab. To increase pore size, a decrease in the mixing ratio of tPEG-SH and tPEG-MA is required, leading to less crosslinking [40]. If the pore size formed in the hydrogel is smaller than the hydrodynamic size of bevacizumab, no drug release at all is possible.

The water content of hydrogels can affect the loading/releasing mechanisms of the drugs through the pores of hydrogel [41]. Thus, we tested the hydrogels in a balanced salt solution designed as an aqueous replacement for intraocular use. Both gels have been designed to have an ultra-low swelling pressure as swelling cannot be tolerated in the eye and have a polymer content of 1% or below with a refractive index close to water indicating a high water content/hydration. Thus, the effect of swelling and thus water content on our results is thought to be tolerable. Further, the pH can also affect drug loading and release. However, the goal of this study was to simulate the clinical situation in the vitreous cavity with a highly controlled pH. Also, all monomers used in our study are highly hydrophilic. Drug loading and release can also be influenced by hydrophobic niches within the gel. However, these should not occur in the proposed gels.

Finally, the stability of the products could be a confounding factor in the differences we found. However, we previously examined the stability of the hydrogels using an accelerated in vitro aging model showcasing good stability [42]. Thus, as the presented experiments only last 24 h, no confounding is to be expected.

## 3. Conclusions

In this study, we analyzed the in vitro pharmacokinetics of bevacizumab in the silicone oil-filled eye in comparison to hydrogel replacements and the PVB. This was tested for various compounds and is in line with in vivo data. For the silicone oils, no uptake of bevacizumab could be shown at all. This translates to a supposed quick washout in vivo with a reduced uptake by the retinal tissue. Additionally, the simultaneous use of bevacizumab and silicone oils is questionable, because of a drastic loss in interfacial tension and an increase in emulsification of silicone oils was observable and emulsification can occur to a greater extent. The hydrogel replacements differed with a 5- (alginate) and 20-fold (tPEG) increased bevacizumab level compared to PVB. A possible reservoir function of the hydrogels needs to be further evaluated. Up to now, no hydrogels for this purpose are commercially available. However, these experiments are sought to illuminate challenges that will occur during clinical practice after regulatory concerns have been addressed.

## 4. Materials and Methods

Bevacizumab (Avastin^®^ 25 mg/mL) was purchased from Roche Pharma AG (Grenzach-Wyhlen, Germany). Balanced salt solution (BSS) was purchased from Beaver-Visitec International Ltd. (Waltham, MA, USA). F6H8, Densiron 68 (D68) and Siluron 5000 (S5000) were provided by Fluoron GmbH (Ulm, Germany). Alginate solution (0.5%) was purchased from Alginatec GmbH (Riedenheim, Germany). Raw materials for the synthesis of the third-generation hydrogel 4ARM-SH-10K (M = 10.000 g/mol) and 4ARM-MA-10K (M = 10.000 g/mol) were purchased from JenKem Technology (Tianjin, China).

### 4.1. Preparation of the Aqueous Phase

In clinical practice, patients receive a single intravitreal injection of 1.25 mg/0.05 mL bevacizumab which results in a starting concentration of 2.27 mg/mL based on the assumptions of the posterior segment model (please see respective paragraph). The aqueous phase was prepared by diluting a stock solution of 25 mg/mL bevacizumab to a concentration of 2.27 mg/mL in BSS.

### 4.2. In Vitro Posterior Segment Model

**Figure 7:**
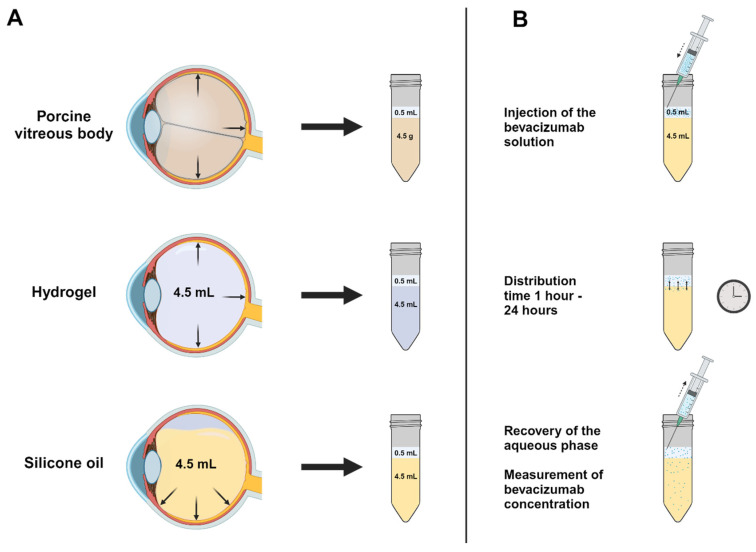
Experimental setup of the in vitro posterior segment model. (**A**) Translation of the posterior segment of the eye into an in vitro posterior segment model. On the top left, the human vitreous body was evaluated, which led to an experimental setup for the hydrogels (middle left) and the silicone oils (bottom left). (**B**) A 0.5 mL amount of the bevacizumab solution was transferred to the vitreous body substitute (shown for the example of silicone oil) and recovered after the respective time.

The goal of this in vitro posterior segment model is to simulate the different volumes in the vitreous cavity after vitreoretinal surgery as well as the eye movements. The model is designed to allow the recovery of the aqueous phase as only the aqueous phase can undergo quantitative measurements. A 4.5 mL amount of the respective vitreous body substitute was transferred into a 15 mL falcon tube. A 500 µL amount of the 2.27 mg/mL bevacizumab solution was injected into the falcon tube and placed on a rocking table at 21 °C for 1, 3, 6, 12, and 24 h. The aqueous phase was recovered and quantified by Nanodrop measurements (Thermo Scientific NanoDrop 2000 UV–Vis Spectrophotometer, Darmstadt, Germany) and ELISA measurements (ProteoGenix, Schiltigheim, France). Densiron 68 and Siluron 5000 were carried out sixfold at each time point. F6H8, alginate hydrogel, and the tPEG hydrogel were carried out threefold and conducted in triplicates. Therefore, each sample was prepared three times individually. Every sample was then tested three times in the assay (Figure 7).

### 4.3. Quantification of Bevacizumab Concentrations

All concentrations of the test samples were quantified using a Nanodrop (Thermo Scientific NanoDrop 2000 UV–Vis Spectrophotometer). Therefore, a serial ten-point (0.05–25 mg/mL) calibration with the clinically used Avastin stock solution of 25 mg/mL bevacizumab was diluted in BSS (R^2^ = 0.9999). The vitreous body loses hyaluronic and other proteins during the study period, making Nanodrop measurements imprecise. Thus, to quantify the concentration of bevacizumab in the porcine vitreous body, an ELISA (ProteoGenix, Schiltigheim, France) was applied. A serial five-point calibration in the range of 78 to 1.250 ng/mL was performed (R^2^ = 0.9955). The recovery of the aqueous phase of the porcine vitreous body was immediately conducted and stored at 8 °C for a maximum of 24 h. The starting concentration was quantified with both, NanoDrop and ELISA to ensure valid concentrations and corresponding calibration. Controls with the starting concentration of bevacizumab were also measured over 24 h to ensure the stability of bevacizumab in the measurement setup.

### 4.4. Synthesis of Hydrogels

#### 4.4.1. Second-Generation Vitreous Body Replacement Hydrogel

As second-generation hydrogel, 4.5 mL of alginate monomers (0.5%, Alginatec, Riedenheim, Germany) were dialyzed (8 kDa, Ø 11.5 mm; Spectra/Por^®^ 7 Dialysis Membrane, Repligen, Boston, MA, USA) in a 11.6 mM calcium sulfate solution for at least 4 h. The alginate hydrogel was removed from the dialysis membrane and rinsed with balanced salt solution before further processing [8,43]. Further chemical details are described by Russo et al. [44].

#### 4.4.2. Third-Generation Vitreous Body Replacement Hydrogel

As third-generation hydrogel, we used a tetra-polyethylene glycol (tPEG) hydrogel joint by Michael addition (click chemistry of thiol and maleimide functions). Therefore, Tetra-PEG-SH and Tetra-PEG-MA (molecular weight 10 kDa) were dissolved in citrate phosphate buffer (CPB: pH 5.0, di-sodium hydrogen phosphate and citric acid monohydrate) in concentrations of 12.6 g/L and 7.4 g/L, respectively. NMR was conducted to further analyze the chemical structure prior commencement of the experiments confirming the monomers’ structure and substitution of greater than 90%. MALDI was conducted prior to substitution to confirm the molecular weight of the PEG structures (molecular weight of the monomers 10,213 and 10,224 Da). Equal volumes of the High-Tetra-PEG solution (12.6 g/L) of one monomer were mixed with the Low-Tetra-PEG solution (7.4 g/L) of the other monomer to generate two oligo-Tetra-PEG hydrogels. After 12 h, equal volumes of the oligo-Tetra-PEG hydrogels were mixed, generating a Tetra-PEG hydrogel [17].

### 4.5. Surface Tension Measurement

A K100C Force Tensiometer (KRÜSS GmbH, Hamburg, Deutschland) was used to measure the interfacial tension in a fully automated fashion. A TJ40 was used to temperate the samples. A Du Noüy Ring (RI01) and the Du Noüy Ring GFS measurement program were used. First, samples were acclimated to 35 °C. To ensure drug stability, this was only conducted immediately before the measurements. Subsequently, the phase with the higher density was placed into the measurement container, then the measuring element was placed. Lastly, the other phase was carefully poured into the measurement container. By lifting the measuring element, the interfacial tension can be calculated. As measurements require roughly 50 mL per phase per measurement, only 4 measurements in total were conducted. Densiron 68 and Siluron 5000 were tested with balanced salt solution with and without the addition of bevacizumab (2.27 mg/mL).

### 4.6. Statistical Analysis

Data are presented in mean ± standard deviation (SD). Prism Version 8.4.0 (GraphPad Software, San Diego, CA, USA) and STATA 17BE (StataCorp, College Station, TX, USA) were used to analyze the respective data. Normal distribution was examined using Kolmogorov–Smirnov test for Densiron 68 and Siluron2000. For comparison, the *t*-test was used as appropriate.

## Data Availability

Data are available from the corresponding author upon reasonable request.
